# The risk-based breast screening (RIBBS) study protocol: a personalized screening model for young women

**DOI:** 10.1007/s11547-024-01797-9

**Published:** 2024-03-21

**Authors:** Gisella Gennaro, Lauro Bucchi, Alessandra Ravaioli, Manuel Zorzi, Fabio Falcini, Francesca Russo, Francesca Caumo

**Affiliations:** 1 Veneto Institute of Oncology (IOV) IRCCS, Padua, Italy; 2 Emilia-Romagna Cancer Registry, Romagna Cancer Institute, IRCCS Istituto Romagnolo per lo Studio dei Tumori (IRST) Dino Amadori, Meldola, Forlì, Italy; 3 SER - Servizio Epidemiologico Regionale e Registri, Azienda Zero, Padua, Italy; 4 Cancer Prevention Unit, Local Health Authority, Forlì, Italy; 5 Direzione Prevenzione, Sicurezza Alimentare, Veterinaria, Regione del Veneto, Venice, Italy

**Keywords:** Breast cancer, Cancer screening, Breast density, Risk assessment, Digital Breast Tomosynthesis

## Abstract

The optimal mammography screening strategy for women aged 45–49 years is a matter of debate. We present the RIBBS study protocol, a quasi-experimental, prospective, population-based study comparing a risk- and breast density-stratified screening model (interventional cohort) with annual digital mammography (DM) screening (observational control cohort) in a real-world setting. The interventional cohort consists of 10,269 women aged 45 years enrolled between 2020 and 2021 from two provinces of the Veneto Region (northen Italy). At baseline, participants underwent two-view digital breast tomosynthesis (DBT) and completed the Tyrer-Cuzick risk prediction model. Volumetric breast density (VBD) was calculated from DBT and the lifetime risk (LTR) was estimated by including VBD among the risk factors. Based on VBD and LTR, women were classified into five subgroups with specific screening protocols for subsequent screening rounds: (1) LTR ≤ 17% and nondense breast: biennial DBT; (2) LTR ≤ 17% and dense breast: biennial DBT and ultrasound; (3) LTR 17–30% or LTR > 30% without family history of BC, and nondense breast: annual DBT; (4) LTR 17–30% or > 30% without family history of BC, and dense breast: annual DBT and ultrasound; and (5) LTR > 30% and family history of BC: annual DBT and breast MRI. The interventional cohort is still ongoing. An observational, nonequivalent control cohort of 43,000 women aged 45 years participating in an annual DM screening programme was recruited in three provinces of the neighbouring Emilia-Romagna Region. Cumulative incidence rates of advanced BC at three, five, and ten years between the two cohorts will be compared, adjusting for the incidence difference at baseline.

*Trial registration* This study is registered on Clinicaltrials.gov (NCT05675085).

## Background

The 2006 European guidelines for mammography screening endorsed organized screening programmes to target women aged 50–69 years [1], a recommendation supported by leading medical agencies [2]. As a result, only a limited number of European countries and regions have adopted organized screening for women aged 40–49 or 45–49 years, usually with a two-year screening interval [3].

In 2017, the European Commission’s Breast Cancer Guidelines Development Group conditionally recommended mammography screening for women aged 45–49 years [4]. However, the optimal screening interval remained undecided, with proposals for biennial or triennial rather than annual screening. The evolving diagnostic landscape has underscored the need to determine the most effective screening frequency.

Exploration of screening intervals for younger women is intertwined with risk-stratified screening, which tailors screening decisions to an individual’s breast cancer risk by considering age at onset, screening frequency, and imaging modality [5]. Breast cancer research has highlighted the different aspects of the disease, which include genotypes, phenotypes, prognosis and therapeutic responses. This shift toward personalized treatment of breast cancer reflects the broader trend of precision medicine, which tailors therapy to individual characteristics [6–8].

Similarly, the traditional “one-size-fits-all” approach to breast cancer screening, which is based solely on the age criterion, has become obsolete because it does not take account of the biologic and epidemiologic complexity of the disease [9, 10]. Breast cancer is the result of a complex web of causation involving multiple interacting risk factors. This has given rise to a debate about risk-based screening protocols [11–16] and to the development of many risk models that incorporate classic risk factors, such as the patient age, family history of breast and ovarian cancer, personal history and reproductive history [17–20]. Over the years, breast density [21] has been incorporated into some risk models [22–24] in order to improve the accuracy of risk prediction. In fact, breast density can mask tumours and increase the interval cancer rates [25], which has suggested the use of supplemental imaging for women with dense breasts in screening programmes [26–28]. In addition, breast density is independent of other risk factors and is associated with increased risk that is not explained by its masking effect [21].

Although the potential benefits of screening stratified by density and risk are promising, two critical key challenges require attention. First, there are significant gaps in our knowledge base, especially regarding the relative effectiveness of different stratified protocols compared with each other and with traditional mammography screening [20, 29, 30]. To answer these scientific questions, a number of randomized controlled trials are underway [16, 31], as well as mathematical modeling studies measuring the effectiveness and cost-effectiveness of different screening frequencies [32–34].

Second, the implementation of screening programmes stratified by breast density and risk faces organizational and resource barriers. These include training, performance, workload, and financial sustainability within the existing healthcare system [35, 36]. Consequently, further observational studies of screening practices stratified by breast density and risk are essential [30].

This article introduces an innovative study protocol that offers a new perspective. Our study employs a quasi-experimental, prospective, population-based approach to evaluate the efficacy and feasibility of a screening model stratified by breast density and risk for women aged 45–49 years. Our goal is to evaluate the impact of this new screening approach in a real-world setting, contrasting it with a standard public health screening regimen. The quasi-experimental design provides an optimal framework for examining both the efficacy and feasibility of this innovative screening method.

## Materials and methods

### Context

In Italy, the public health system offers mammography screening every two years for women aged 50–69 years. Conversely, only a few regions, notably Emilia-Romagna, have implemented annual mammography screening for women aged 45–49 years [37, 38]. In contrast, the Veneto Region, like most Italian regions, does not yet have a mammography screening programme for women aged 45–49 years, despite the fact that breast cancer incidence rates are comparable to those observed among women aged 50–54 years [39, 40].

In 2018, the Veneto Institute of Oncology (IOV) addressed this gap by proposing a project to the Veneto Region, aiming to explore the feasibility and sustainability of a personalized screening programme. This innovative approach incorporates tomosynthesis, individual breast density, and risk estimation to personalize screening of women aged 45–49 years. The Regional Administration approved a feasibility study focusing on 45-year-old women residing in the provinces of Padua and Rovigo, totaling about 10,000 women per year. Given the limited pool of eligible participants, a quasi-experimental approach was adopted. In this setup, IOV formed the prospective cohort, while the observational control cohort was enrolled from the Emilia-Romagna Region.

### Study design

The study uses a quasi-experimental design of pre-post comparison with a non-equivalent control group [41]. Quasi-experimental designs, wich are used when random intervention assignment is impractical or unethical, reflect real-world conditions and allow for generalization. Despite practicality, cost-effectiveness, and higher external validity, this design may suffer from lower internal validity due to systematic differences between interventional and control groups. Nonetheless, non-experimental designs have been already used to assess the impact of public health mammography screening on the incidence of advanced breast cancer [42, 43].

We recruited a prospective interventional screening cohort of 45-year-old women from two provinces of the Veneto Region. These women, after initial screening, were divided into five subgroups based on their breast density and individual risk and were referred for different screening intervals and imaging protocols until the age of 49 years. At the same time, we assembled an observational cohort from the Emilia-Romagna Region, composed of women of the same age who underwent standard annual mammography screening. This population will serve as a nonequivalent control for comparative purposes.

The study hypothesis is that a screening model stratified by breast density and risk is more effective and sustainable in reducing the incidence of advanced breast cancer than standard annual mammography screening.

Both the interventional and control cohorts are studied using a two-phase design involving a pre-screening period followed by a screening period. The pre-screening phase measures the baseline incidence rate of breast cancer in women aged 45–49 years in both populations. This baseline assessment helps calculate the ratio of the incidence rate at baseline between the Veneto Region and the Emilia-Romagna Region for subsequent statistical adjustment. During the screening period, the two screening models are implemented, and cumulative incidence rates of advanced breast cancer are assessed over 3 years, 5 years and 10 years (at least). The ratio of the cumulative incidence rate in the interventional cohort to that found in the observational control cohort, adjusted for differences in incidence at baseline, is calculated. The decision to evaluate the results at three-year intervals stems from previous research that demonstrated a significant decline in the incidence of pT2-4 stage breast cancer from the third year of screening [44]. The extended follow-up duration takes into account the potential lasting preventive impact of screening beyond age 50 and incorporates the common biennial digital mammography screening procedure for women over age 50.

### Interventional cohort

The prospective interventional cohort consists of women aged 45 years from two provinces in the Veneto Region. The enrollment phase for this cohort has been completed, with a final number of participants of 10,269 women. Participants initially met specific criteria and are now undergoing successive screening rounds with different intervals and imaging protocols until the age of 50.

During the initial screening round, participants underwent two-view digital breast tomosynthesis (DBT) of both breasts. They also completed a questionnaire on risk factors such as personal characteristics, hormonal factors, family history of breast cancer, lifestyle, and health status. Volumetric breast density (VBD) values were calculated with Volpara software from DBT images [45]. Subsequently, women with an average VBD greater than 25% were invited for supplemental ultrasound (US). Based on individual risk factors and mean VBD, we used the Tyrer-Cuzick risk model to estimate lifetime risk (LTR) for each woman [17, 22]. This risk assessment categorized women into five subgroups each recommended for a personalized screening protocol:

1. *Low-Risk, Nondense Breasts*: Women with LTR ≤ 17% and VBD < 25% are recommended for biennial DBT screening;
2. *Low-Risk, Dense Breasts*: Women with LTR ≤ 17% and VBD ≥ 25% are recommended for biennial DBT screening with supplemental US (DBT + US protocol);
3. *Intermediate-Risk, Nondense Breasts*: Women with LTR between 17 and 30%, or LTR > 30% without family history, and VBD < 25% are recommended for annual DBT screening;
4. *Intermediate-Risk, Dense Breasts*: Women with LTR between 17 and 30%, or LTR > 30% without family history, and VBD ≥ 25% are recommended for annual DBT + US screening;
5. *High-Risk, Any Breast Density*: Women with LTR > 30% and a family history of breast cancer, are recommended for annual DBT and magnetic resonance imaging (MRI) surveillance, regardless of breast density.

These individualized recommendations based on density and risk offer personalized screening protocols. For low to intermediate risk nondense breasts, DBT examinations were/are performed during initial and subsequent rounds and interpreted independently by two breast radiologists. For dense breasts at low or intermediate risk, DBT and US are performed by a single breast radiologist. Abnormal findings require further evaluation, including additional imaging and, if necessary, biopsy. Post-treatment follow-up plans are initiated for confirmed breast cancer cases. Informed consent was obtained by all women participating in the interventional cohort.

### Observational control cohort

Between 1995 and 1997, the Emilia-Romagna Region started a double-reading mammography screening programme for women aged 50–69 years, which switched from analog to digital mammography (DM) in 2010–2011. The programme was extended to women aged 45–49 years and 70–74 years in 2010, with an annual screening interval for the younger age group [46]. In the context of the RIBBS study, the observational control cohort includes women 45 years of age residing in three provinces of the Emilia-Romagna Region. These individuals participated in the annual screening programme from 2012 to 2020.

**Fig. 1:**
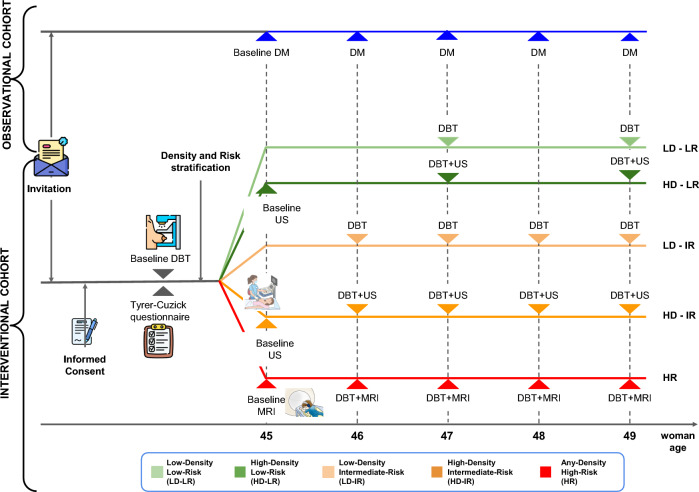
Technical scheme depicting the five subgroups of the interventional cohort and the observational control cohort of the Risk-Based Breast Screening (RIBBS) study

The five subgroups of the interventional cohort and the observational control cohort are depicted in Fig. 1.

### Pre-screening and screening periods

The pre-screening phase for the interventional cohort ran from 2017 to 2019, while that of the control population ran from 2005 to 2009. Subsequently, the screening phase started in 2020 for the interventional cohort and 2012 for the control population. The screening phase will end in 2026 for the interventional cohort and 2024 for the observational cohort. These timelines are designed to ensure that all women who entered the screening programme at age 45 are screened until they turn 50.

### Study hypothesis

The interventional cohort is stratified into five subgroups based on breast density and individual risk, each associated with an imaging protocol expected to have greater sensitivity than mammography in the control cohort. The two-year screening interval for the two low-risk interventional subgroups (with low and high density, respectively) compared with the control cohort (annual mammography) does not denote de-escalation, as digital breast tomosynthesis remains more sensitive than mammography. In brief, the study hypothesis predicts the superiority of the personalized screening approach in decreasing the cumulative incidence of advanced breast cancer compared with the conventional “one-size-fits-all” mammography screening procedure.

### Outcome measures

The primary and secondary outcome measures are listed in Table 1. The primary measure of the effectiveness of screening stratified by breast density and risk, compared with conventional annual mammography, will be the cumulative incidence of advanced breast cancer. In particular, the focus will be on stage II or higher cancer throughout the follow-up period. Most of the secondary outcome measures are part of the core indicators used in the annual national survey of regional mammography screening programmes in Italy. These indicators were defined by the Italian Group for Mammography Screening and the Italian National Centre for Screening Monitoring [48]. They constitute the benchmark for reporting outcomes of breast cancer screening programmes in Italy.

**Table 1:** Primary and secondary outcome measures in the RIBBS study

	Description
Primary outcome meausure: cumulative incidence of advanced breast cancer	Measure of effectiveness of screening stratified by breast density and risk compared with standard annual digital mammography. Focuses on stage II or higher cancer throughout the follow-up period
Secondary outcome measures	
Recall rate	Number of women recalled for further diagnostic evaluation per thousand women undergoing each of the two screening protocols
Cancer detection rate	Number of women with breast cancer detected by screening (topographic code C50 [47]) per thousand women undergoing each of the two screening protocols
Proportional incidence of interval cancer	Number of women diagnosed with interval cancer (detected after a negative screening episode) divided by the expected number of breast cancer cases in the absence of screening, by screening protocol and interval year
Total assessment rate	Number of women undergoing diagnostic evaluation per thousand women undergoing each of the two screening models, further subdivided into non-invasive and invasive assessment rates
Surgical referral rate	Number of women referred for excisional biopsy or definitive surgical treatment per thousand women undergoing each of the two screening protocols
Surgery rate	Number of women undergoing excisional biopsy or definitive surgical treatment per thousand women undergoing each of the two screening protocols
Benign lesion detection rate	Number of women with any histologically diagnosed benign lesion per thousand women undergoing each of the two screening protocols
Tumour-stage specific detection rate	Number of women with cancer detected by screening and classified by TNM tumour stage per thousand women undergoing each of the two screening protocols
Regular re-screening rate	Number of women who regularly undergo the specific screening protocol (within ± 3 months) between ages 45 and 49 per thousand women in the interventional and control groups. Calculated for the total interventional cohort and the observational control cohort

Table 2 provides an overview of the additional analyses planned as part of the RIBBS study.

**Table 2:** Additional analyses planned as part of the RIBBS study

Analysis definition	Description
Cost and organizational impact analysis	Examines both the direct costs associated with implementing and operating the stratified screening programme, as well as the economic feasibility and financial implications of adopting the personalized approach. Evaluates expenses related to screening equipment, staff training, data management systems, and potential changes in workflow and resource allocation within screening units. Provides insights for policy makers, healthcare administrators, and stakeholders involved in decision-making regarding breast cancer screening strategies
Impact of breast cancer risk model on personalization of screening	Compares the proportions of women categorized as low, intermediate, and high risk for breast cancer using different risk models (Tyrer-Cuzick, Gail, Boadicea) to assess risk stratification implications in personalized screening
Impact of breast density metrics on personalization of screening	Compares the proportions of women with dense and non-dense breasts using various breast density metrics (Volumetric Breast Density, Area-Based Breast Density, BI-RADS category) to evaluate breast density stratification implications in personalized screening
Impact of different breast density metrics on breast cancer risk assessment	Compares proportions of women classified as low, intermediate, and high risk using the Tyrer-Cuzick risk model when different breast density metrics are utilized. Examines implications of different density measures for risk stratification in personalized screening
Prevalence analysis of breast cancer subtypes	Evaluates the distribution by breast cancer subtype (luminal A, luminal B, HER2-positive, basal-like) across the overall study population and when stratified by breast density and risk category. Analyses subtype prevalence variations according to breast density and individual risk profiles
Potential of artificial intelligence (AI) to supportpersonalization of screening	Explores AI integration into personalized screening protocols. Assesses potential reduction in readers needed for personalized protocols with double reading and workload reduction from AI's accurate classification of clearly negative exams. Evaluates benefits like cost savings, efficiency, resource utilization, radiologist productivity, and job satisfaction. Measures AI effectiveness in different subgroups based on breast density and risk categories

### Inclusion criteria

The criteria for inclusion into the interventional and observational cohorts are shown in Table 3.

**Table 3:** Inclusion criteria for the interventional and observational cohorts of the RIBBS study

	Interventional cohort	Observational cohort
Female	X	X
45-year-old	X	X
Resident in defined provinces of the two regions	X	X
Willing and able to give written informed consent	X	NA
Willing and able to comply with scheduled visits, tests, and other procedures	X	NA

NA, Not Applicable. In the interventional cohort, ethics committee approval and the signing of an informed consent form by each participating woman was required. The observational cohort is derived from a population-based screening programme, in which participation is considered indicative of consent

### Exclusion criteria

The exclusion criteria for both cohorts are shown in Table 4.

**Table 4:** Exclusion criteria for the interventional and observational cohorts of the RIBBS study

	Interventional cohort	Observational cohort
Recent mammography	X	X
Personal history of invasive breast cancer and ductal carcinoma in situ		
	X	X
Known or suspected germline mutation of BRCA1/2, PALB2, TP53 or equivalent	X	X
Psychiatric and other disorders not compatible with compliance to the protocol requirements and follow-up	X	X
Pregnancy or breastfeeding	X	X
Unable to give informed consent	X	NA
Current participation in another breast screening trial	X	NA

NA: Not Applicable. In the interventional cohort, ethics committee approval and the signing of an informed consent form by each participating woman was required. The observational cohort is derived from a population-based screening program, in which participation is considered indicative of consent

### Data collection and management

Data collection and management will adhere to general Italian data protection regulations, ensuring maximum privacy and security of participants' information.

For the interventional cohort, a database dedicated to the RIBBS study was developed. This repository includes several tables containing essential information, such as mean VBD, calculated with the Volpara software, individual risk factors included in the IBIS software for lifetime risk assessment based on the Tyrer-Cuzick risk model, recall data derived from double readings, and details obtained from any required diagnostic procedure, including histologic data from biopsies. The raw data will be accessible for a variety of analyses or subanalyses.

As regards the observational control cohort, primary records will be obtained from the Emilia-Romagna Region mammography screening data warehouse. These data provide comprehensive information on invitations, mammograms, diagnostic procedures for abnormal screening outcomes, and benign or malignant lesions detected by screening. The case series is linked with the population-based regional breast cancer registry to obtain additional information.

To identify incident cases of breast cancer, all women in the interventional and observational control cohorts will be linked to the Veneto and Emilia-Romagna Cancer Registries, respectively. Automated case registration through the Veneto Cancer Registry has recently demonstrated a high level of consistency and reliability [49]. Previous studies have also validated the record-linkage protocols in both registries [38, 50]. Primary information available from both registries includes sociodemographic characteristics, primary cancer site, TNM tumour stage, histologic type, and vital status.

### Sample size

Sample size calculations focused specifically on the primary outcome measure of the personalized screening procedure. This primary outcome measure revolves around assessing the reduction in the incidence of advanced breast cancer in the interventional cohort compared with the observational control cohort.

Considering the real-world context of the study and the constraints associated with enrolling participants in both cohorts, the sample size calculations encountered some limitations. The enrollment of patients in both cohorts was limited by factors such as available space and tight timelines due to the initial approval period granted by the Veneto Region. This consequently prevented expansion of the cohorts beyond the approved period.

Given the impossibility of accurately determining the sample size required to detect a specific effect on the primary outcome with the desired statistical power, our approach involved estimating the minimum detectable reduction in the incidence of advanced BCs using the sample size provided and ensuring a minimum power of 80%. The sample sizes available to conduct a one-sided test comparing the null hypothesis H0: rate ratio ≥ 1.000 and the alternative Ha: rate ratio < 1.000, using the W5 Variance Stabilized test statistic, were determined in 43,000 subjects in the observational control cohort and 10,269 subjects in the total interventional screening cohort, both over a 5-year exposure period. With these designated sample sizes, the study is able to achieve a power of 80.5% in detecting an event rate ratio under the alternative hipothesis (RRa) of 0.810. The event rate in the observational control cohort (λ1) is estimated to be 0.004017, based on the incidence rate of BC at baseline. Specifically, the significance level (alpha) used in the test is 0.050.

### Statistical analysis

Regarding the primary objective of the study, which focuses on evaluating the impact of the personalized screening model on the cumulative incidence of advanced breast cancer (stage II or higher), a series of analyses are planned. Person-years at risk and follow-up duration will be calculated for both cohorts, starting from the date of first screening and continuing until the date of censoring or the end of follow-up, whichever comes first. The follow-up duration will be truncated at the 75th percentile of available follow-up times, as determined by the criterion proposed by Puliti et al. [51].

Woman age will be calculated at the date of the first screening. Cumulative age-standardized advanced breast cancer incidence rates will be calculated for both cohorts and then compared using the incidence rate ratio (IRR) along with 95% confidence interval (95% CI). Estimation of IRRs will be obtained by multivariable Poisson regression analysis.

To address potential dissimilarities (if any) in the risk of breast cancer between the two cohorts, incidence rates from the pre-intervention period will be used. Adjusting the IRR of advanced breast cancer will involve multiplying the unadjusted IRRs by the observed/expected ratio within the observational control cohort. Calculation of 95% CIs will be performed by a bootstrap procedure.

### Dissemination plan

The results of the study will be widely disseminated through rigorous publications in reputable peer-reviewed journals. In addition, results will be communicated through presentations at regional, national, and international workshops and conferences. The dissemination strategy will primarily target clinicians, researchers and crucial stakeholders, including scientific societies and the Italian regional healthcare authorities. To ensure transparency and adherence to reporting standards, the study will adhere to the 'REporting of studies Conducted using Observational Routinely collected health Data' (RECORD) guidelines.

## Discussion

This study represents a novel exploration into the impact of a personalized screening strategy, tailored according to breast density and risk, on the cumulative incidence of advanced breast cancer and other standard screening performance metrics among young women.

The study is particularly relevant because of the growing consensus on lowering the age of entry in breast cancer screening to 45 years and the ongoing worldwide debate about moving from a uniform age-based model to personalized, woman-centered approaches.

A key strength of this project is the innovative approach to personalizing breast cancer screening. A screening programme was developed for the interventional cohort, taking into account both breast density and cancer risk, two critical factors in personalizing screening. For women with dense breast, the inclusion of ultrasound complements annual or biennial DBT to mitigate the limitations of X-ray imaging. In addition, the screening interval for low-risk women has been increased. Stratification of density and risk is based on objective data, including volumetric breast density and lifetime risk derived from relevant risk factors.

In summary, we believe that the methods employed provide a valid and pragmatic means of evaluating the effectiveness and feasibility of a screening procedure stratified by breast density and risk in real-world settings.

### Study limitations

Due to practical and ethical concerns, a quasi-experimental design was adopted for this study. The nonrandomized nature of our design introduces potential challenges to internal validity because of discrepancies between the nonequivalent control group and the interventional group. In addition, contextual factors may affect the expected outcome [42]. However, to address potential bias from the nonrandomized design, a comparison between the two cohorts will involve adjustment for the incidence of breast cancer observed during the pre-intervention period [42, 51, 52].

Another limitation of the study is the absence of clinical and epidemiologic data on women in the reference population. This lack of data precludes a formal adjustment for potential confounding factors.

### Study status

In the Veneto Region, enrollment in the interventional cohort began in January 2020 and ended in December 2021, successfully enrolling a total of 10,269 women aged 45 years. Currently, these participants are regularly invited to participate in subsequent screening rounds according to the established schedule. The screening phase for this cohort will extend until December 2026, providing full coverage until women reach age 50. Thereafter, they will enter the target population of the standard two-year mammography screening programme, with routine invitations until age 69.

The observational control cohort comprises women aged 45 years who were enrolled in the annual mammography screening programme between 2012 and 2020 in three provinces of the Emilia-Romagna Region, for a total number of 43,840 women. As in the Veneto Region, when these women cross the 50-year threshold, they will become part of the population eligible for biennial mammography screening.

Follow-up evaluations will continue for at least 10 years after the enrolment in the study.

## Data Availability

Not applicable.

## Abbreviations

BC: Breast cancer
BI-RADS: Breast imaging reporting and data system (American College of Radiology)
BMI: Body mass index
CI: Confidence interval
DBT: Digital breast tomosynthesis
DM: Digital mammography
H0: Null hypothesis
Ha: Alternative hypothesis
IOV: Veneto Institute of Oncology
IRR: Incidence rate ratio
LTR: Lifetime risk
MRI: Magnetic resonance imaging
RIBBS: Risk-based breast screening
RRa: Event rate ratio under the alternative hypothesis
US: Ultrasound
VBD: Volumetric breast density
